# Real-Time and Long-Term Effects of Medical Marijuana on Older Adults: Protocol for a Prospective Cohort Study

**DOI:** 10.2196/78900

**Published:** 2026-03-24

**Authors:** Kendall R Robinson, Stella D Seeger, Lauren Nave, Marlin Mejia, Maria Vander Meulen, Ahmed Rashid, Angela M Mickle, Kimberly T Sibille, Zhigang Li, Rene Przkora, Siegfried Schmidt, Margaret C Lo, Robert L Cook, Yan Wang

**Affiliations:** 1 Department of Epidemiology, University of Florida, CTRB Bldg, 4th Fl., 2004 Mowry Rd, Gainesville, FL, 32610, United States, 1-352-273-7920; 2 McCombs School of Business, University of Texas, Austin, TX, United States; 3 Miller School of Medicine, University of Miami, Miami, FL, United States; 4 College of Medicine, University of Florida, Jacksonville, FL, United States; 5 Department of Anesthesiology, University of Florida, Gainesville, FL, United States; 6 Department of Physical Medicine and Rehabilitation, University of Florida, Gainesville, FL, United States; 7 Department of Biostatistics, University of Florida, Gainesville, FL, United States; 8 Department of Community Health and Family Medicine, College of Medicine, University of Florida, Gainesville, FL, United States; 9 Department of Medicine, Division of General Internal Medicine, College of Medicine, University of Florida, Gainesville, FL, United States

**Keywords:** medical marijuana, MM, cannabis, older adults, chronic pain, ecological momentary assessment, EMA

## Abstract

**Background:**

Older adults represent the fastest-growing group of medical marijuana (MM) users in the United States, with chronic pain being the most common reason for use. Despite this trend, scientific evidence remains limited regarding the short- and long-term effects of MM on critical health outcomes, including cognitive function, physical and mental health, and overall quality of life, in this population. To better inform clinical practice and public policy, there is a clear need for more rigorous longitudinal studies that examine the impact of real-world MM products over time.

**Objective:**

The Study on Medical Marijuana and Its Long-Term Effects on Older Adults (SMILE) is a prospective cohort study that aims to (1) determine MM’s short- and long-term effects on pain, physical, emotional, and cognitive functioning, as well as quality of life, in older adults and (2) identify MM product characteristics and patient subgroups associated with improved outcomes and side effects.

**Methods:**

This study will recruit and follow 440 older adults (aged ≥50 years; ~50% aged >65 years, ~50% male) with chronic pain for 12 months, as some initiate MM use (MM group, n=330) and others do not (comparison group, n=110). Data collection includes quarterly survey questionnaires (longitudinal changes in cannabis use, pain, physical and emotional functioning, side effects, and quality of life); baseline and 12-month cognitive assessments, pain sensory tests, and blood and urine samples for cannabis use; and periodic smartphone- and Fitbit sensor–based measurements to capture MM use patterns, real-time pain, mental health, and objective data on physical activity and sleep. Data will be analyzed using descriptive analyses, generalized linear mixed-effects models, and generalized estimating equation models to assess differences in short- and long-term effects between the MM and comparison groups, as well as subgroups among those initiating MM treatment.

**Results:**

Recruitment for the SMILE study began in July 2022, and all data collection is expected to be completed by 2026. As of October 2025, we have enrolled 399 participants, with 277 in the MM group and 122 in the comparison group. Results are expected to be published starting in 2027.

**Conclusions:**

With multisource data collected in real time and over 12 months, our study will provide much-needed scientific evidence addressing (1) whether MM can reduce pain and improve physical and emotional functioning in the short term among older adults; (2) whether the effects of MM last for 12 months and demonstrate changes in quality of life or cognition; and (3) whether health benefits and consequences differ by MM product type and whether individual differences (eg, sex, baseline pain phenotyping) moderate the relationship. Our findings will offer valuable insights for physicians and patients when considering MM as a treatment option and will help guide more informed, individualized care decisions.

## Introduction

Medical marijuana (MM) is increasingly accessible in the United States. Its use is rising among the 36 million adults in the United States aged 45 years or older, with chronic pain accounting for 60.6% of qualifying conditions for MM use [1-4]. This increase in MM use could be attributed to state-level legalization of marijuana and older adults seeking MM as an alternative pain treatment, which reduces stigma and increases accessibility [5-7]. However, evidence on marijuana’s long-term efficacy and safety in older adults is scarce [1,8-21], as most studies only include participants under the age of 60 [22]. This is concerning, given the association of aging with polypharmacy and substantial changes in pharmacokinetics and pharmacodynamics (eg, drug clearance rate) [5,23]. Therefore, data on short- and long-term health outcomes and side effects among older adults are urgently needed [24].

While marijuana’s pharmacological profile supports a potential role in pain management, empirical evidence regarding its efficacy in treating chronic pain remains inconsistent. While some meta-analyses have reported significant beneficial effects of marijuana on chronic pain [25-27], others have found no or limited effect [28-30]. Similarly, the literature has shown that MM may help with chronic pain-related experiences—such as anxiety, depression, and opioid use—while also improving sleep and quality of life among individuals with chronic pain [31-34]. Despite the significant interpersonal differences in responses to pain treatment [35], important biological factors (eg, sex and baseline pain phenotyping), psychological factors (eg, marijuana expectancy and social desirability bias), and the use of objective measures such as sensors or biomarkers to complement self-reported outcomes [36-38] have received limited attention in marijuana and chronic pain research [22,39,40].

Research challenges—such as a reliance on the use of marijuana products not reflective of real-world usage of MM—may explain the inconsistent findings across studies [41]. While randomized controlled trials are considered the “gold standard” of evidence, the products used are often isolated or synthetic cannabinoids (eg, dronabinol) [42,43], which have limited variety and may not be comparable to MM products available in state dispensaries [32]. The ubiquitous access to MM at the state level dictates an urgent need to investigate the effects of these products, which usually have higher concentrations of tetrahydrocannabinol (THC) and/or cannabidiol (CBD) and are consumed via a greater diversity of methods (eg, vape, capsule, tincture, cream). The lack of standard guidance for MM use makes it difficult to accurately characterize the ratio of major components (THC:CBD), administration route, and dosage, all of which may influence the efficacy of MM on chronic pain [1,44,45]. There remains a critical need for systematic investigation of MM’s effects on pain, physical and emotional functioning, and overall health outcomes (eg, quality of life), as well as the need to track potential side effects, such as its impact on cognitive functioning in this population [22,46,47].

To address these critical gaps in the literature, this study will build a prospective cohort of older adults aged 50 years or older with chronic pain, including those who initiate MM use and those who do not. This study will integrate both technology-based remote assessments (eg, smartphone-based ecological momentary assessment [EMA], Fitbit sensor) and quarterly in-person visits (eg, surveys, blood tests) to systematically examine the short- and long-term impacts of MM on 6 core chronic pain outcome domains: (1) pain intensity and interference; (2) physical functioning; (3) emotional functioning; (4) cognitive function; (5) participant-rated overall improvement; and (6) adverse events or side effects [48-50], 5 of which are recommended by the Initiative on Methods, Measurement, and Pain Assessment in Clinical Trials (IMMPACT).

The specific aims of this project are as follows: (1) determine whether MM use leads to short-term changes in pain intensity and physical and emotional functioning, as measured in real time by EMA (subjective data) and the Fitbit device (objective data); (2) evaluate whether MM use leads to longer-term changes over a 12-month period, based on self-reported pain intensity or interference, emotional and physical functioning, health-related quality of life, and cellular aging markers, such as telomere length; and (3) among individuals initiating MM, examine which MM product characteristics (ie, THC:CBD ratio, administration route, dose) are associated with greater improvements in the outcomes defined in Aims 1 and 2, or with a greater incidence of side effects. Additionally, the study will explore whether individual differences (eg, sex, baseline pain phenotype) moderate these relationships.

## Methods

### Ethical Considerations

The University of Florida (UF) Institutional Review Board (IRB) approved this study (IRB202101597) on July 29, 2021, and it was conducted in accordance with the Declaration of Helsinki.

All participants will provide written informed consent prior to any study procedures being conducted, with the option to withdraw consent at any time. The study team will send participants the informed consent form remotely through a secure Research Electronic Data Capture (REDCap) link for review before their baseline visit. Participants have the option to sign the consent form remotely or after discussing study details with study staff during the baseline visit. Following informed consent, participants will complete the Rapid Estimate of Adult Literacy in Medicine, Revised to assess health literacy [51]. If participants score lower than 6 out of 8 points on the Rapid Estimate of Adult Literacy in Medicine, Revised, study staff will re-review the informed consent forms and ask follow-up questions to ensure that participants fully understand the study protocol.

Only IRB-approved study staff will have access to study information and participant data. Participants are assigned a unique study identifier to maintain confidentiality. Biospecimens are labeled with the unique study identifier, sample type, study time point, and date of collection. Whether a participant is in the MM group or the comparison group will not be disclosed to prevent bias during sample processing. All survey data will be kept in the secure REDCap system during the data collection phase. National Institutes of Health (NIH) Toolbox data will only contain the study ID as an identifier and will be emailed with encryption to the data manager for upload into REDCap. EMA data will only contain the study ID as an identifier and will be downloaded from cloud storage to a secured server at UF on a biweekly basis when active data collection is in process. Any photos of MM products (participants are instructed to avoid including any personal identifiers in the photo) uploaded by study participants during EMA surveys will be stored on a secured server. Fitbit data will be downloaded from a Fitbit database service (ie, Fitabase) via encrypted transmission. Research staff will use the unique study account to match it back to the participant’s unique study ID so the Fitbit data can be merged with other data collected from the same participant. A certificate of confidentiality was automatically granted by the NIH to protect participant privacy because the study is funded by the NIH.

Participants will be compensated for their participation, including US $60 for completing the baseline visit, US $40 for each in-person quarterly follow-up visit, and US $60 for the 12-month follow-up visit. Participants are compensated up to US $280 for EMA and Fitbit and are paid in full if they complete 80% of the EMA assessments and wear their Fitbit 80% of the time during each period. If they do not reach this threshold, prorated payments are provided. Travel compensation of an additional US $20 is provided for participants who travel 20 or more miles for the baseline and 12-month visits. Remote options for 3-, 6-, and 9-month visits are offered for individuals experiencing transportation barriers. An additional US $200 payment is provided for participants in the MM group to offset initial costs associated with beginning MM treatment if they delay initiating MM treatment for at least 3 days to provide clean baseline data (without cannabis use) for EMA and Fitbit and then obtain an MM card through the state of Florida. Participants may keep the Fitbit device as part of their compensation after the 12-month follow-up visit or return it if they do not want it. Participants are compensated or given study merchandise of equal monetary value (~US $10, eg, umbrella, mug, hand sanitizer) if they fail screening procedures after enrollment.

### Participants

#### Population

This study plans to recruit 440 older adult participants aged 50 years or older, with at least 50% over the age of 65, and with approximately 50% of the sample being male. We will try to achieve the balanced ratio by monitoring sample demographics throughout the study and adjusting recruitment techniques as needed to target necessary demographics. Among these, 330 participants will be seeking to start MM treatment during the study period, as indicated at baseline when they are enrolled in the study, and 110 participants will have no intention of starting MM treatment for at least 6 months. However, participants are free to initiate or stop MM use at any time during the study and continue participation after they join the study. If a participant decides to withdraw from the study or is lost to follow-up at any point, data will be analyzed up to the point of withdrawal.

#### Inclusion Criteria

Potential participants will be eligible for the Study on Medical Marijuana and Its Long-Term Effects on Older Adults (SMILE) if they (1) are 50 years or older; (2) have had noncancer chronic musculoskeletal or neuropathic pain [52] for 3 or more months [53], with a pain intensity level in the past week greater than zero (“0=no pain” to “10=most pain imaginable”) [54]; (3) are seeking MM treatment (for the MM group) or have no intention to start MM for at least 6 months (for the comparison group); and (4) have access to a smartphone.

Individuals aged 50 years and older were chosen due to the rising use of MM for chronic pain and the need for research on this population [5,6]. Participants must have current noncancer chronic musculoskeletal or neuropathic chronic pain, confirmed by verification of pain for 3 or more months at an intensity level greater than zero. Intention to initiate MM treatment is assessed at recruitment to determine which group enrolled participants will be in. To obtain baseline data before the initiation of MM treatment, participants must not be receiving MM treatment at the time of enrollment. Individuals who are not interested in MM should ideally not initiate MM treatment within the 6 months following enrollment. Access to a smartphone is necessary for completing the EMA and Fitbit aspects of the study. Because all assessments and questionnaires that will be used are in English, participants must be able to read English to complete study measures.

#### Exclusion Criteria

Potential participants will be excluded if they (1) are currently pregnant or nursing (by self-report); (2) self-report marijuana or CBD use in the past 30 days, or if the urine test is positive for THC; (3) report any history of using MM regularly (daily use); (4) used marijuana regularly (daily use) for over a month in the past 10 years; (5) have current or past substantial or severe substance use disorders [55], as assessed by the Drug Abuse Screening Test [56,57]; (6) are undergoing active cancer treatment, excluding certain cancers such as skin cancer; (7) have a terminal illness; (8) have neurological conditions that impact their ability to consent or complete study procedures (eg, Alzheimer disease, Parkinson disease, multiple sclerosis, head injury, stroke, and/or seizures); or (9) have active, uncontrolled psychiatric disorders (eg, schizophrenia, bipolar disorder, excluding anxiety and depression). Many of these exclusion criteria are imposed to ensure participant safety, the ability to provide consent, and the completion of study procedures.

### Recruitment and Consent

The primary recruitment method for both MM and comparison groups will be through UF’s health care system contact registry (Consent2Share), where patients have consented to be contacted for research. The research team will first contact patients with chronic pain in the contact registry via email or messages within the MyChart patient portal; those who are interested will then be prescreened for preliminary eligibility over the phone. The UF Pain Research and Intervention Center of Excellence (PRICE) registry (IRB201400126) will also be used to recruit participants who have previously consented to be contacted about future research studies.

Additional participant recruitment will take place at various chronic pain, primary care, and MM clinics across the state of Florida, as well as the Veterans Affairs (VA) Medical Center. The research coordinator or staff has a designated space at clinics to determine preliminary eligibility, explain details of the study, and schedule an in-person baseline visit. Clinic staff or clinicians will distribute study flyers and contact forms to their patients and waiting areas. The VA coordinators will also prescreen potential research participants via VA medical records or physician referrals and contact potential participants via mail, email, or phone calls. Clinics may also send electronic versions of IRB-approved flyers and recruitment materials to their scheduled patients or direct patients to fill out an online form to express interest in the study. We will recruit in community settings via flyers in research facilities and clinics and through radio advertising. All assessments will be conducted at research facilities in Gainesville or Jacksonville, Florida.

### Study Design

#### Overview

**Figure 1.**
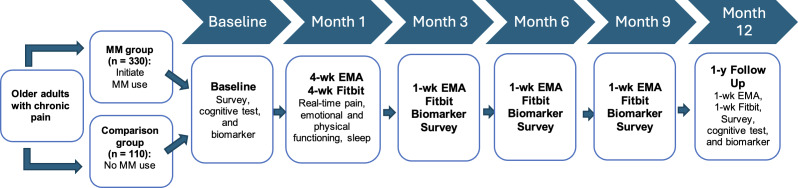
Study procedure overview. EMA: ecological momentary assessment; MM: medical marijuana.

All participants will complete an in-person screening, including a urine sample to confirm their marijuana use status, verification of musculoskeletal or neuropathic chronic pain using medication bottles or medical records, and a screening questionnaire. Participants will complete in-person assessments at baseline and 12 months, including survey questionnaires, cognitive testing, blood pressure measurement, and biomarker collection (blood samples for cannabis metabolite tests, telomere length, and inflammation/inflammasome markers). After the initial baseline visit, participants will complete technology-based real-time assessments using a smartphone-based EMA app to measure subjective elements of pain intensity, emotional and physical functioning, and sleep. Participants will also be provided with a wearable Fitbit sensor to record objective data on heart rate, physical activity, and sleep. The EMA surveys and Fitbit data will capture real-time short-term effects during the first month and for 1 week every 3 months over 12 months. Biospecimen collection (urine tests and blood samples) and a follow-up survey will be repeated every 3 months. Participants are encouraged to provide feedback regarding study procedures throughout their participation to inform future revisions to the protocol. Figure 1 provides an overview of the study procedures. A detailed timeline for each measure and assessment conducted throughout the course of the study can be found in Table 1.

**Table 1.** Summary of study measures and assessments.

Measure	Baseline	3, 6, and 9 months	12 month	Source
Screening				
Presence of tetrahydrocannabinol (THC) in urine	✓	✓^a^	✓^a^	Urine test
Current/history of substance use disorders	✓			DAST-10^b^ [56,57]
Health literacy	✓			REALM-R^c^ [51]
Biometric data/biospecimen				
Height and weight	✓		✓	N/A^d^
Blood pressure	✓	✓	✓	N/A
Telomere length	✓		✓	Blood sample: buffy coat [58-60]
Cannabinoids and metabolites	✓	✓	✓	Blood sample: cannabinoids/metabolites assay [61-63]
Inflammation and neurodegeneration	✓	✓	✓	Blood sample: inflammasome and neurodegeneration biomarker test (exploratory)
Pain sensory tests				
Pain sensitivity	✓		✓	Punctate mechanical stimuli test and pressure pain threshold [64]
NIH^e^ Toolbox Cognition Battery				
Executive function	✓		✓	NIH Toolbox dimensional change card sort test and NIH Toolbox Flanker inhibitory control and attention test [65]
Processing speed	✓		✓	NIH Toolbox pattern comparison processing speed test [65]
Episodic memory	✓		✓	NIH Toolbox picture sequence memory test [65]
Working memory	✓		✓	NIH Toolbox list sorting working memory test [65]
Language	✓			NIH Toolbox picture vocabulary test and NIH Toolbox oral reading recognition test [65]
Other assessments				
Real-time outcomes (pain, mood, sleep) and medication/MM^f^ use	4 wk	1 wk	1 wk	EMA^g^ smartphone app and Fitbit; BSI^h^ [66]

a Urine test at follow-up does not affect eligibility.

b DAST-10: Drug Abuse Screening Test.

c REALM-R: Rapid Estimate of Adult Literacy in Medicine, Revised.

d N/A: not applicable.

e NIH: National Institutes of Health.

f MM: medical marijuana.

g EMA: ecological momentary assessment.

h BSI: Brief Symptom Inventory.

#### Group Assignment

Participants will be assigned to the MM group or the comparison group based on their responses to baseline screening questions regarding current interest or disinterest in starting MM treatment. Group assignment will remain consistent for each participant, regardless of whether they change their mind throughout the course of the study. Participants interested in initiating MM treatment will be given information about local MM physicians who can certify them for a Florida MM card. Additional compensation for delaying MM treatment to enroll in the study will also be provided to participants who begin MM treatment within 4 weeks after their baseline visit to cover most of the cost for MM physician visits and the initial charge to obtain their MM card in Florida.

#### Surveys at Baseline and 3-, 6-, 9-, and 12-Month Follow-Ups

Surveys at all time points take approximately 30 to 45 minutes for participants to complete. The length of the visit can be a barrier for older adults, especially those with chronic pain. In the interest of saving participants’ time and preventing fatigue during the in-person assessment, participants will have the option to complete the survey remotely before their visit. Participants are offered snacks and beverages during in-person visits and can take as many breaks as needed. The survey includes questions such as demographics, health status measured using the Charlson Comorbidity Index and health history [67], medication and supplement use measured using the Medication Quantification Scale, patient-reported medication symptoms adapted for MM, nonpharmacological treatments [68-70], self-rated health, mental well-being, history of and current nonmedical marijuana use, and substance use. Established scales will be used to assess variables such as pain measured by the Brief Pain Inventory, Graded Chronic Pain Scale (GCPS), Patient Global Impression of Change, and Patient-Reported Outcomes Measurement Information System (PROMIS) Pain Interference Scale [71-74]; marijuana use measured by the comprehensive marijuana motives questionnaire adapted by Lankenau et al [75-78], Medical Cannabis Expectancy Questionnaire, and Cannabis Use Disorders Identification Test-Revised; substance use measured by the World Health Organization-Alcohol, Smoking and Substance Involvement Screening Test [79]; quality of life measured by the PROMIS Global-10 [80]; depression and anxiety measured by the PROMIS Anxiety and Depression Scales [81]; social desirability measured by the Marlowe-Crowne Social Desirability Scale [82]; post-traumatic stress disorder measured by the Primary Care-Post-Traumatic Stress Disorder Scale [83]; and sleep quality measured by the Pittsburgh Sleep Quality Index [84]. A full list of core survey assessments can be found in Table 2, and information on how these measures are related to each study aim is outlined in the *Analytical Strategy* section. Participants will complete the survey independently or with assistance from research staff.

**Table 2.** Summary of measures included in the core and quarterly follow-up surveys.

Measure	Baseline	3, 6, and 9 months	12 month	Source
Demographics, income, and insurance	✓			N/A^a^
Medical history	✓	✓	✓	CCI^b^ and health history [67]
Current medication use	✓	✓	✓	MQS^c^ [68]
Marijuana use				
Marijuana use motive	✓			CMMQ^d^—adapted by Lankenau et al [75,78]
MM^e^ attitudes and perceptions	✓		✓	N/A
Positive and negative MM expectations	✓			MCEQ^f^ [76]
Cannabis use disorders	✓		✓	CUDIT-R^g^ [77]
List of MM products^h^		✓	✓	N/A
Recreational marijuana use	✓	✓	✓	N/A
Marijuana-related adverse events		✓	✓	PRMS^i^—adapted for MM^h^ [70]
Pain				
Pain assessment	✓	✓	✓	BPI^j^ [71]
Pain severity	✓		✓	GCPS^k^ [72]
Change in pain			✓	PGIC^l^ [73]
Chronic pain stage	✓		✓	Pain FITT^m^ [37,58,85]
Pain interference	✓	✓	✓	PROMIS^n^ Pain Interference Scale-Version 4a [74]
Nonpharmacological treatments	✓	✓	✓	Nonpharmacological therapies for chronic pain [69]
Emotion				
Depression	✓	✓	✓	PROMIS Depression Scale-8a [81]
Anxiety	✓	✓	✓	PROMIS Anxiety Scale-8a [81]
Health-related quality of life	✓	✓	✓	PROMIS Global-10 [80]
Social desirability	✓			MCSDS^o^ [82]
PTSD^p^	✓			4-item PC-PTSD^q^ Scale [83]
Other				
Sleep	✓	✓	✓	Pittsburgh Sleep Quality Index [84]
Substance use	✓		✓	WHO-ASSIST^r^ [79]

a N/A: not applicable.

b CCI: Charlson Comorbidity Index.

c MQS: Medication Quantification Scale.

d CMMQ: Comprehensive Marijuana Motives Questionnaire.

e MM: medical marijuana.

f MCEQ: Medical Cannabis Expectancy Questionnaire.

g CUDIT-R: Cannabis Use Disorders Identification Test-Revised.

h Administered to the Medical Marijuana group only.

i PRMS: patient-reported medication symptoms.

j BPI: Brief Pain Inventory.

k GCPS: Graded Chronic Pain Scale.

l PGIC: Patient Global Impression of Change.

m Pain FITT: pain frequency, intensity, time, and total number of pain sites.

n PROMIS: Patient-Reported Outcomes Measurement Information System.

o MCSDS: Marlowe-Crowne Social Desirability Scale.

p PTSD: post-traumatic stress disorder.

q PC-PTSD: Primary Care PTSD.

r WHO-ASSIST: World Health Organization-Alcohol, Smoking, and Substance Involvement Screening Test.

Follow-up surveys will be conducted at 3, 6, 9, and 12 months after the baseline assessments. Quarterly follow-up surveys at 3, 6, and 9 months will collect information on MM products (if applicable), current medications, and any health updates. At the 12-month visit, participants will complete the same core survey as the baseline, excluding demographic questions. Follow-up surveys may be completed remotely.

#### Biospecimens

At every visit, participants will be asked to provide a urine sample to test for THC. For remote follow-up visits, a drug test cup will be mailed with instructions on how to upload a photo of the results.

We will also collect approximately 4 mL of blood every 3 months from each participant completing an in-person visit. Plasma will be shipped to the University of Colorado laboratory for analysis of various cannabinoids and associated metabolites (6a-OH-CBD, 7-OH-CBD, 11OH-THC, THC, CBD, CBC, CBD-gluc, THC-gluc, 7-CBD-COOH, THC-COOH, THC-COOH-Gluc, CBN, CBG, THCV, CBDV) [61-63]. Backup plasma samples may also be shipped to the University of Miami for inflammasome and/or neurodegeneration biomarker tests for exploratory analysis.

For the blood drawn at baseline and 12-month visits, the buffy coat will be frozen for analysis of telomere length, a biological measure of cellular aging [58-60]. Collected samples will be sent to the Blackburn Lab at the University of California, San Francisco, for analysis at the end of the study.

#### Anthropometric and Cardiovascular Assessments

Participant’s height and weight will be measured at the baseline and 12-month study visits. Every 3 months, study staff will measure the participant’s blood pressure using a vital sign monitoring device during in-person visits.

#### Clinical and Experimental Pain Measures at Baseline and 12-Month Visits

Clinical pain measures will include the GCPS [72], which assesses chronic pain severity, and the Chronic Pain Stage, which is a stage measure based on pain frequency, intensity, time, and total number of pain sites [37,58,85]. Experimental pain measures will include punctate mechanical stimuli and pressure pain threshold to assess pain sensitivity, which has shown predictive utility for pain severity in chronic pain patients [64].

#### Standardized Neurocognitive Assessments at Baseline and 12-Month Visits

After completing the baseline and 12-month follow-up surveys, participants will complete cognitive assessments using the NIH Toolbox Cognition Battery, a standardized neurocognitive assessment tool available as an iPad app [65]. It contains 7 cognition battery tests assessing 5 cognitive subdomains: Language, Executive Function, Episodic Memory, Processing Speed, and Working Memory [65]. Table 1 shows the full list of assessments used. Three composite scores are created from these tests (Fluid, Crystallized, Total Composites), with normative standards available for comparison [86,87]. At the 12-month follow-up visit, only the fluid composite score will be used.

#### EMA Survey

Participants starting MM treatment will complete a 1-week EMA using a smartphone app (mEMA, illumivu Inc. or MetricWire) before they start MM and a 3-week EMA after they begin using it. They will also complete a 1-week EMA at 3, 6, 9, and 12 months to track their MM use and longitudinal real-time outcomes. The comparison group will complete an approximately 4-week EMA at baseline and an approximately 1-week EMA at 3, 6, 9, and 12 months to answer questions regarding medication and nonpharmaceutical use.

This study will use 4 random prompts (roughly 1 every 4 hours) to assess real-time pain levels, mood (measured by the Brief Symptom Inventory[66]), and other symptoms (eg, nausea, sleepiness, dizziness) and 1 daily prompt to assess overall pain levels in the past 24 hours, the previous night’s sleep quality, and medications taken in the past 24 hours besides MM. After the baseline survey and cognitive assessment, study staff will train participants on how to use the EMA with practice questions. Participants starting MM will also be asked to upload a picture of the label on the MM products they have been using to validate the product information.

#### Fitbit Activity Tracker

Participants will also wear a Fitbit Charge 5 or 6 (the latest model available) in parallel with EMA for objective measures of physical activity, heart rate, and sleep duration and quality. Data will be accessed via Fitabase, a service that allows centralized management of Fitbit data.

#### Quality Assurance

The research team will not be permitted to recruit participants or conduct visits until they receive training and pass evaluation by the study coordinators or principal investigator (PI). EMA and Fitbit data will be assessed multiple times per week to monitor for any missing data or issues with participant compliance. Weekly meetings with the research team and biweekly meetings with the investigators will be held to discuss any protocol deviations or discrepancies. The research coordinators and data manager will conduct biweekly checks of all survey data and visit log notes for discrepancies.

### Analytical Strategy

#### Power and Sample Size

The total sample size was calculated based on Aims 1 and 2, for which statistical analysis will use a “difference-in-differences” approach. Power was calculated to ensure the detection of differences between the baseline and 12-month follow-up survey in Aim 2, which requires a larger sample size than the intensive longitudinal data from EMA assessments. Assuming a 3:1 ratio of MM versus comparison group and within-subject correlation of variables of 0.5, the sample size required to detect an effect size of 0.3 SD between-group difference (small to medium effect size) [88] in the outcome measured by standardized scales (eg, reduction in pain intensity measured by GCPS), with 80% power (type I error=0.05), is 352 (264 vs 88). Assuming 20% attrition, based on our pilot study (79% retention without a preplanned 12-month follow-up), the final sample size required to achieve the power is 440 (330 vs 110) [89]. For Aim 1, we will have more than 80% power to detect an effect size of 0.3 SD in between-group difference because there will be more repeated measurements in Aim 1. For Aim 3, we will have 264 participants for the analysis since only the MM group will be analyzed. After adjusting for multiple testing with the conservative Bonferroni method [90], the adjusted type I error rate becomes 0.05/7=0.007, under which we will have 99% power to detect the Pearson correlation of 0.3.

#### Aim 1

Aim 1 is to determine whether MM use leads to short-term changes in pain intensity and physical and emotional functioning, as measured in real time by EMA (subjective data) and the Fitbit device (objective data).

Key outcomes of interest include pain intensity, physical functioning, emotional functioning, and sleep. Pain intensity will be assessed from the EMA surveys using self-reported real-time pain intensity on a 0 to 100 visual analog scale. Physical functioning will be assessed from the EMA surveys using self-reported real-time pain interference and from the Fitbit using step counts. Emotional functioning will be assessed from the EMA surveys using self-reported real-time anxiety and depression symptoms (Brief Symptom Inventory).

Separate generalized linear mixed-effects models (GLMM) or generalized estimating equations (GEE) will be used to compare the temporal changes in each of the main outcomes (pain intensity level, physical and emotional functioning) between the MM and comparison groups. A difference-in-difference analysis will be conducted. A propensity score method will account for potential baseline differences between the 2 groups for analysis with inverse probability of treatment weighting. Daily MM use measured via EMA will be a time-varying exposure variable in the GLMM or GEE models to account for crossover between groups. The research team will also include time-varying covariates in these models to account for potential impacts of other non-pharmaceutical pain treatments (eg, category of treatments and number of sessions for each category) and non-MM use (self-reported frequency/quantity).

#### Aim 2

Aim 2 is to evaluate whether MM use leads to longer-term changes over a 12-month period based on self-reported pain intensity, emotional and physical functioning, health-related quality of life, and cellular aging markers such as telomere length.

Key outcomes of interest include pain intensity and interference, physical functioning, emotional functioning, cognitive function, opioid, benzodiazepine, and other medication use, sleep quality, quality of life, and cellular aging. Pain intensity will be assessed from EMA surveys, similar to Aim 1, as well as Brief Pain Inventory and GCPS responses from the core survey. Physical functioning will be assessed from EMA surveys, similar to Aim 1, and the PROMIS Pain Interference Scale from the core survey. Emotional functioning will be assessed from EMA surveys, similar to Aim 1, and the PROMIS Anxiety and Depression Scales from the core survey. Cognitive function will be assessed using the NIH Toolbox Cognition Battery and self-reported changes in cognition from the core survey. Opioid, benzodiazepine, and other medication use will be assessed using the Medication Quantification Scale from the core survey. Sleep quality will be assessed using self-reported sleep duration and quality from the EMA surveys, the Pittsburgh Sleep Quality Index from the core survey, and Fitbit-recorded sleep duration. Quality of life will be assessed using the PROMIS Global-10 and Patient Global Impression of Change from the core survey. Cellular aging will be assessed by measuring telomere length from buffy coat samples.

The research team will analyze 2 types of data: (1) intensive longitudinal data (EMA, Fitbit, opioid, and benzodiazepine dose) and (2) data collected only at baseline and 12 months (core survey, cognitive tests, biomarkers). For intensive longitudinal data, separate GLMM or GEE models will be built to compare longitudinal trajectories in each outcome (pain intensity, emotional and physical functioning, opioid dose) measured at multiple time points over 12 months between the MM group and the comparison group. As in Aim 1, we will treat MM use as a time-varying exposure variable to account for crossover and include other nonpharmaceutical pain treatments and nonmedical marijuana as time-varying covariates. The analysis will use the exact time (eg, hours) of each measurement (instead of days) for constructing the trajectory and thus does not need to average measurements within each day into a single value. For measures collected only at baseline and 12 months, generalized linear regression models will be used to compare group differences in the survey data (eg, anxiety/depression, disability, quality of life), cognitive test results, and telomere length. Additionally, milligram morphine equivalent opioid dosage from the prescription drug monitoring program will be obtained through medical records to assess differences in the risk of unintentional overdose between the MM and comparison groups.

#### Aim 3

Aim 3 is to examine among individuals initiating MM which MM product characteristics (ie, THC:CBD ratio, administration route, dose) are associated with greater improvements in the outcomes defined in Aims 1 and 2, or with a greater incidence of side effects, and explore whether individual differences (eg, sex, baseline pain phenotype) moderate these relationships. Key outcomes of interest are defined in Aims 1 and 2.

Separate GLMM or GEE models using each product characteristic as a predictor will be developed. As in Aims 1 and 2, we will treat MM use as a time-varying exposure variable to account for crossover and include other nonpharmaceutical pain treatments and nonmedical marijuana as time-varying covariates. The research team will construct a multivariate model to include all 3 product characteristics (ie, THC:CBD ratio, administration route, dose) as predictors for each main outcome using stepwise model selection or least absolute shrinkage and selection operator to account for potential interactions within these product characteristics. To determine significant moderating effects of interpersonal differences, separate GLMM or GEE models will be conducted by gender (male vs female) or at each level of the moderator (low/moderate/high stage of pain, low/high marijuana expectancy, low/high social desirability bias) to assess whether the main effect of the MM product characteristic on each main outcome differs by group membership or the level of the moderator.

#### Missing Data

Missing data will be examined to determine whether information is missing at random by comparing participants with and without missing data. If no significant differences are observed in key demographics, missing at random will be assumed. If missingness exceeds 5%, we will use multiple imputation methods to impute missing responses. Sensitivity analyses will be conducted to assess the impact of the imputed data.

### Possible Risks and Discomforts

#### Adverse Events

Adverse events and discomforts may occur due to participation in the study. In the event of adverse events, the PI will determine whether the event is serious and related to the study procedures. If this is the case, the PI will report the event to the NIH and IRB within 72 hours of the event. If the event is not serious, it will be reported to the NIH and IRB during regular status reports.

#### Breach of Confidentiality

Our study will collect data on participants’ medications, including MM. Breaches of confidentiality could occur through communications with participants, loss of research data, or transfer of data.

#### Questionnaires and Interviews

The assessments used in this study address some sensitive issues, such as mental well-being and drug use. The major disadvantages of these assessments are the time required to complete them and the potential breach of confidentiality. Our past experience with these measures indicates that they are acceptable to participants.

#### Cognitive Tests

There are minimal risks associated with these tests. Participants may experience fatigue and feelings of frustration while completing the cognitive function tests. Some participants may also experience stress associated with being tested, though this tends to be quite limited.

#### EMA Surveys

The EMA involves minimal risk as it only collects self-reported data related to participants’ symptoms and their relief, experienced side effects, and other related feelings (eg, anxiety, depression). The major disadvantage of this assessment is the time required to complete daily reports. The possibility of a confidentiality breach is very low because the data collected from the mobile app will be encrypted and pushed to the cloud-based storage database. No data are ever stored on the server’s file system but always in the database. Access to the database is gated, so entry is only permitted to users entering through the approved route (ie, you cannot hack your way into the database by guessing the URL).

#### Pain Sensory Tests

Procedures may be uncomfortable or unpleasant. Participants may experience some temporary discomfort from the pressure and mechanical pain testing. However, if they feel the pain is greater than they wish to tolerate, they can stop any of the procedures at any time.

#### Urine Sample Collection

The risk is minimal for urine sample collection, although some people may experience embarrassment when providing the sample.

#### Blood Collection

The risks of drawing blood from a vein include discomfort at the site of puncture; possible bruising and swelling around the puncture site; rarely, an infection; and uncommonly, faintness from the procedure.

#### Fitbit Data Collection

The risk is minimal when wearing a Fitbit during the EMA period. All data from Fitbit do not include personal identifiers. Data will be stored temporarily on cloud-based storage and will be downloaded regularly onto a secure UF server. Data will be encrypted during the transmission process to ensure security.

#### Anthropometric and Cardiovascular Assessments

The risk is minimal for collecting these measures, including height, weight, blood pressure, and heart rate. Some participants may feel slightly uncomfortable or embarrassed about having some of these measures taken.

## Results

The SMILE study was funded by the NIH/National Institute on Aging in February 2022. Recruitment for the SMILE study began in July 2022 and is expected to continue until 2025, with the last participants recruited completing study involvement in 2026. As of October 2025, we have enrolled 399 participants, with 277 in the MM group and 122 in the comparison group. Data analysis is currently underway, and results are expected to be published starting in 2027. The anticipated timeline for the study is shown in Table 3.

**Table 3.** Study timeline.

Research tasks	2022		2023		2024		2025		2026	
	H1^a^	H2	H1	H2	H1	H2	H1	H2	H1	H2
Recruitment and baseline assessments		✓	✓	✓	✓	✓	✓	✓		
Follow-up assessments (EMA^b^/Fitbit, 1-y visit)			✓	✓	✓	✓	✓	✓	✓	✓
Data/merge analysis and results dissemination				✓	✓	✓	✓	✓	✓	✓

a H1 and H2 indicate the first and second halves of the year.

b EMA: ecological momentary assessment.

## Discussion

### Strengths and Innovation

This project is the first prospective cohort study to comprehensively investigate the IMMPACT-recommended core outcomes [48-50] and cognitive function, using multisource (survey, EMA, sensor, and biomarker) subjective and objective data collected in real time and over 12 months to evaluate the effects of MM use on older adults with chronic pain. This project is innovative in a number of ways.

### Use of Smartphone and Sensor-Based EMA

Mobile technology has rapidly revolutionized health care and research, but it has not yet been widely applied in efforts to investigate the emerging phenomenon of widespread MM use among older adults [45]. This project is the first to leverage both smartphone-based EMA and Fitbit devices to obtain detailed MM use pattern data, as well as subjective and objective real-time measures of pain and functioning in patients’ daily lives. Such data can also provide valuable insights into which MM products are associated with improvements in short-term outcomes [91, 92].

### Enhanced Longitudinal Cohort Design

Our study is the first to leverage a measurement burst design within a cohort study to examine the short- and longer-term effects of MM on older adults. The baseline and 12-month follow-up data will provide comprehensive assessments of pain, functioning, and overall quality of life as snapshots at 2 time points, whereas the quarterly EMA measurement bursts will provide detailed data on short-term variability in pain intensity and physical and emotional functioning, as well as the trajectory of change over 12 months. We will include baseline assessments before participants initiate MM treatment and a comparison group who share similar demographic and health characteristics to reach causal inference.

### Incorporation of Telomere Length as a Measure to Assess MM’s Effect on Cellular Aging Over a 12-Month Period

We and others have shown that telomere length, a measure of cellular aging, is a biomarker of stress-related burdens and buffers [37,38,59,93]. However, no research has investigated how MM may influence cellular aging, as measured by telomere length, in individuals with chronic pain. The proposed study will address this gap by collecting and analyzing telomere length at baseline and after 12 months.

### Consideration of Interpersonal Differences, Including Pain Phenotyping at Baseline

Our study will be the first to examine whether MM’s effects differ based on biological factors (sex and pain phenotyping) and psychological factors (expectancy and social desirability) using established scales [58,76,82]. Such information has valuable implications for physicians and patients regarding which patient subgroups may experience greater benefits from MM use.

### Inclusion of Blood Tests for Multiple Cannabinoids and Associated Metabolites

Our study is the first to test plasma-level cannabinoids and associated metabolites over the course of 1 year for a cohort of older adults with chronic pain. Previous studies have shown that blood tests of cannabinoids and their metabolites can provide valuable information on marijuana use [61-63]. The rich information obtained from repeated blood tests offers objective measures of cannabis exposure to inform how various compounds (eg, THC, CBD) in MM impact health outcomes in this population.

This study will provide valuable scientific evidence to inform physician and patient decisions on using MM as an alternative or supplemental pain treatment option for older adults. With multisource data collected in real time and over 12 months, our results will contribute to the greatly needed evidence on: (1) whether MM can reduce pain and improve physical and emotional functioning in the short term among older adults; (2) whether the effects of MM last for 12 months and demonstrate quality of life improvements; (3) whether health benefits or consequences differ by MM product type; and (4) which subgroups of patients may benefit more from MM use. Should this study confirm that MM helps to improve pain, emotional and physical functioning, and quality of life without significantly reducing cognitive function over a 12-month period, future steps will involve conducting a more focused investigation of specific MM products (eg, sublingual drops with a 1:1 THC:CBD ratio) that are associated with more beneficial outcomes. Future studies are needed to examine treatment side effects as well as optimal dosage to inform more specific recommendations for MM.

### Limitations

There are several limitations to our study. Patients can obtain an MM card as early as the same day they meet with a qualifying physician, which may limit the ability to enroll participants prior to the initiation of MM treatment. Conversely, some participants in the MM group, despite expressing an intention to start treatment promptly, may experience delays in initiation due to various barriers, such as financial constraints or access issues. To address this issue, we have implemented additional compensation for participants who delay the initiation of their MM treatment to enroll in the study and begin MM treatment within 4 weeks of their baseline visit. Another potential limitation of the SMILE study is the wide variety of MM products and modes of administration available. As this is an observational study, no limitations are placed on what types of products may be used by participants. Attrition bias is a concern, as study participants who experience side effects or receive no benefits from MM treatment may withdraw from the study, which could result in a more favorable outcome toward MM improving chronic pain. To prevent this, our team emphasizes to the participants that we would like to continue monitoring their health outcomes regardless of whether they continue using MM. Our team may also waive certain data measures (eg, EMA, Fitbit) to make participation easier for individuals who are considering withdrawing from the study. Additionally, individuals with chronic pain may be experiencing pain due to undiagnosed cancer, which could result in misclassification.

### Dissemination Plan and Future Research

Results from this study will be published in peer-reviewed research journals and presented at conferences. Authorship for all manuscripts and presentations will follow the International Committee of Medical Journal Editors recommendations. Future research will be needed to expand the literature on the use of MM in older adults with chronic pain. In addition, the use of randomized controlled trials to control for the types of MM products used by participants will be useful in the future.

## Supplementary material

10.2196/78900 Checklist 1 SPIROS checklist.

## Abbreviations

CBD: cannabidiol
EMA: ecological momentary assessment
GCPS: Graded Chronic Pain Scale
GEE: generalized estimating equations
GLMM: generalized linear mixed-effects models
IMMPACT: Initiative on Methods, Measurement, and Pain Assessment in Clinical Trials
IRB: Institutional Review Board
MM: medical marijuana
NIH: National Institutes of Health
PI: principal investigator
PRICE: Pain Research and Intervention Center of Excellence
PROMIS: Patient-Reported Outcomes Measurement Information System
REDCap: Research Electronic Data Capture
SMILE: Study on Medical Marijuana and Its Long-Term Effects on Older Adults
SPIROS: Standardized Protocol Items Recommendations for Observational Studies
THC: tetrahydrocannabinol
UF: University of Florida
VA: Veterans Affairs
